# Levobupivacaine Administration Suppressed Cell Metabolism in Human Adenocarcinoma A549 Cells

**DOI:** 10.3390/ijms262210833

**Published:** 2025-11-07

**Authors:** Masae Iwasaki, Makiko Yamamoto, Masahiro Tomihari, Kaori Fujii, Masashi Ishikawa

**Affiliations:** Department of Anesthesiology and Pain Medicine, Graduate School of Medicine, Nippon Medical School, 1-1-5, Sendagi, Bunkyo 113-8602, Tokyo, Japan; m-yamamoto@nms.ac.jp (M.Y.); m-tomihari@nms.ac.jp (M.T.); kaori-fujii@nms.ac.jp (K.F.); masashi-i@nms.ac.jp (M.I.)

**Keywords:** levobupivacaine, lung adenocarcinoma, ACE2, HIF-1α, Wnt1 pathway, MMP-9

## Abstract

Perioperative anesthesia might directly alter cancer cell biology. We investigated the effects of levobupivacaine treatment on lung adenocarcinoma cells. A549 cells were treated with levobupivacaine at concentrations of 0.1 mM and 0.5 mM for 2 h. Transfection with angiotensin-converting enzyme 2 (ACE2) small interfering RNA (siRNA) was performed 6 h before the levobupivacaine treatment. Cell proliferation was assessed using a cell counting kit 8 (CCK-8), and ATP synthesis was evaluated with the CellTiter-Glo^®^ 2.0 assay at 0 and 24 h after anesthesia exposure. RT-PCR was performed to examine various biomarkers. The levobupivacaine treatment suppressed ATP synthesis without affecting cell proliferation. This was associated with the upregulation of ACE2 and the downregulation of pro-cancer biomarkers, including HIF-1α, MMP-9, and β-catenin. The anticancer effect of levobupivacaine was negated when ACE2 siRNA was introduced, and it was further suppressed when combined with levobupivacaine. The RT-PCR results indicated that the expressions of B-cell/CLL lymphoma 2 (BCL2) and wingless/integrated 1 (WNT1) were reduced after levobupivacaine treatment, but these effects were reversed with ACE2 siRNA induction. The administration of levobupivacaine suppressed A549 cell metabolism and downregulated HIF-1α, MMP-9, WNT1, EGFR, and BCL2 in an ACE2-dependent manner.

## 1. Introduction

Malignant tumors are prevalent and are the primary cause of mortality globally [1]. Lung cancer is the most frequently diagnosed malignant tumor, with approx. 2,400,000 new cases reported each year worldwide [1]. Among the different histological variants, lung adenocarcinoma constitutes ~60% of lung cancer cases [2]. Surgical intervention, along with preoperative and postoperative chemotherapy, is often the standard initial treatment for various cancers. The anesthetics used during surgical procedures have been observed to influence postoperative cancer recurrence and the effectiveness of chemotherapy through multiple mechanisms [3]. Levobupivacaine and ropivacaine are commonly administered local anesthetics. Both are long-acting amide-type anesthetics that inhibit sodium channel activity. Levobupivacaine is recognized as the S-enantiomer of bupivacaine, and ropivacaine is a derivative of bupivacaine [4]. Levobupivacaine and ropivacaine are amino-amide local anesthetics that are optically pure S-enantiomers of bupivacaine. They differ in the length of the N-alkyl side chain, with levobupivacaine containing a butyl group and ropivacaine containing a propyl group [5]. Levobupivacaine is expected to have an equivalent duration of action compared to ropivacaine. Our prior research demonstrated that ropivacaine has a direct inhibitory effect on the proliferation of lung adenocarcinoma cells, mediated by the angiotensin-converting enzyme 2 (ACE2) receptor [6]. ACE2 has been identified as an anticancer factor in lung cancer [7,8,9]. We conducted the present study to (*i*) investigate the direct effects of the local anesthetic levobupivacaine on cultured human lung adenocarcinoma cells and (*ii*) elucidate the molecular biological mechanisms of levobupivacaine, using ACE2 siRNA for further analysis.

## 2. Results

### 2.1. Levobupivacaine Inhibited the Cellular Activity of A549 Cells Through Its Interaction with ACE2

#### 2.1.1. Levobupivacaine Treatment Did Not Alter the A549 Cells’ Proliferation, but ACE2 Knockdown Promoted the Cells’ Proliferation

A549 cells were treated with each concentration of levobupivacaine with/without siRNA transfection. The treatments were as follows. Control (C): no medication but with siRNA transfection, L0.1: levobupivacaine at 0.1 mM for 2 h, L0.5: levobupivacaine at 0.5 mM for 2 h, and si: siRNA transfection for 6 h prior to levobupivacaine treatment.

**Figure 1:**
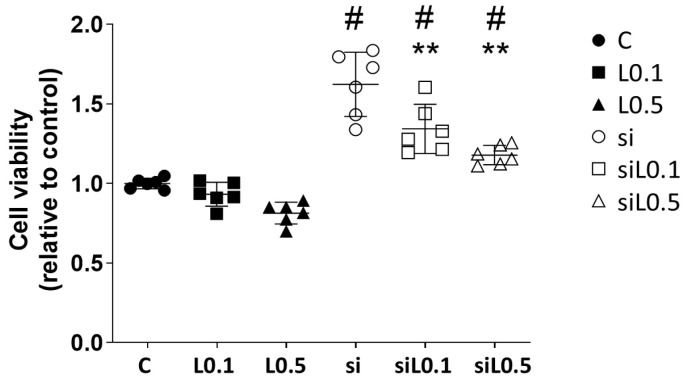
The A549 cell proliferation analysis using CCK8 at 24 h after 2 h of levobupivacaine treatment with/without ACE2 siRNA transfection. ** *p* < 0.01 vs. the C group; # *p* < 0.05 vs. the siRNA-untreated group; *n* = 6, one-way ANOVA followed by a post hoc Tukey test. Dots: no administration of levobupivacaine. Squares: 0.1 mM levobupivacaine. Triangles: 0.5 mM levobupivacaine. Black: no siRNA transfection. Black and white: with siRNA transfection. C: control (scrRNA-treated), L: levobupivacaine, CCK8: cell count kit 8.

Figure 1 illustrates the effects of levobupivacaine treatment on the proliferation ability of A549 cells. Notably, ACE2 knockdown en-hanced the cells’ proliferation; however, the simultaneous administration of levobupi-vacaine inhibited this proliferation (all of the results are presented as the mean ± SD, relative ratio to C, *n* = 6; C 1.000 ± 0.033 vs. si 1.623 ± 0.202 vs. siL0.1 1.344 ± 0.155 vs. siL0.5 1.179 ± 0.061; C vs. si, *p* < 0.0001; si vs. siL0.1, *p* = 0.0028; si vs. siL0.5, *p* < 0.0001; L0.1 vs. siL0.1, *p* < 0.0001; L0.5 vs. siL0.5, *p* < 0.0001).

#### 2.1.2. Levobupivacaine Treatment Resulted in the Suppression of ATP Synthesis, Which Was Reversed by the ACE2 Knockdown

**Figure 2:**
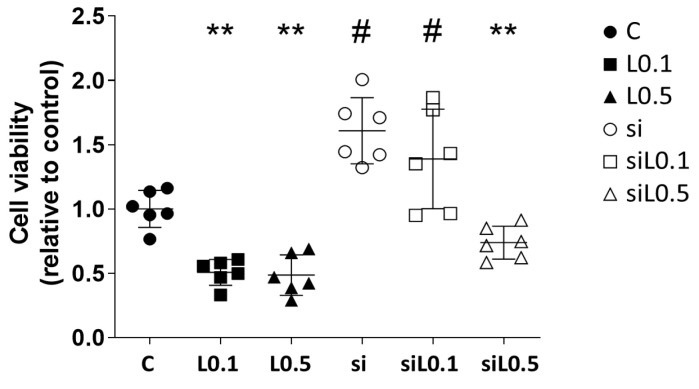
The A549 ATP synthesis analysis using CellTiter-Glo^®^ 2.0 at 24 h after 2 h of levobupivacaine treatment with/without ACE2 siRNA transfection. ** *p* < 0.01 vs. the C group; # *p* < 0.05 vs. the siRNA-untreated group; *n* = 6, one-way ANOVA followed by a post hoc Tukey test. Dots: no administration of levobupivacaine. Squares: 0.1 mM levobupivacaine. Triangles: 0.5 mM levobupivacaine. Black: no siRNA transfection. Black and white: with siRNA transfection. C: control (scrRNA-treated).

Figure 2 illustrates the effects of levobupivacaine treatment on the production of ATP in A549 cells. The treatment with levobupivacaine resulted in a significant reduction in ATP metabolic activity (relative ratio to C, *n* = 6; C 1.000 ± 0.139 vs. L0.1 0.502 ± 0.101 vs. L0.5 0.515 ± 0.179; C vs. L0.1, *p* = 0.0060; C vs. L0.5, *p* = 0.0039; L0.1 vs. L0.5, *p* > 0.9999). The knockdown of ACE2 enhanced the ATP metabolic activity in A549 cells; however, when levobupivacaine was administered concurrently, a suppression of this activity was observed (relative ratio to C, *n* = 6; C 1.000 ± 0.139 vs. si 1.720 ± 0.248 vs. siL0.1 1.337 ± 0.357 vs. siL0.5 0.795 ± 0.118; C vs. si, *p* = 0.0005; si vs. siL0.1, *p* = 0.5234; si vs. siL0.5, *p* < 0.0001; L0.1 vs. siL0.1, *p* < 0.0001; L0.5 vs. siL0.5, *p* = 0.3672).

### 2.2. mRNA Changes with Levobupivacaine and ACE2 siRNA Administration in A549 Cells

#### 2.2.1. Levobupivacaine Treatment Led to an Increase in ACE2 mRNA Expression, Whereas the Administration of siRNA Significantly Reduced the Expression of ACE2

**Figure 3:**
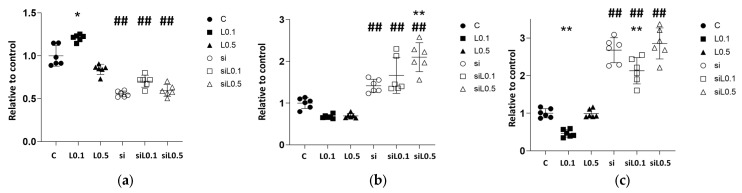

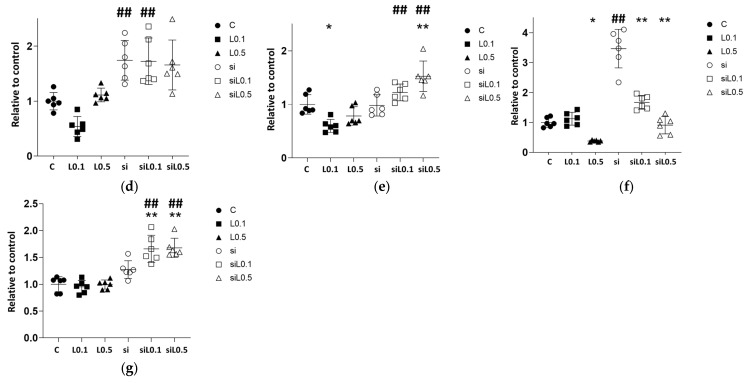
The qRT-PCR results at 6 h after 2 h of levobupivacaine treatment with/without ACE2 siRNA transfection. (**a**) ACE2, (**b**) HIF-1α, (**c**) BCL2, (**d**) EGFR, (**e**) BAX, (**f**) WNT1, and (**g**) β-catenin. Data are mean ± SD. * *p* < 0.05, ** *p* < 0.01 vs. the C group; ## *p* < 0.01 vs. the siR-NA-untreated group; *n* = 6, one-way ANOVA followed by a post hoc Tukey test. C. Dots: no administration of levobupivacaine. Squares: 0.1 mM levobupivacaine, Triangles: 0.5 mM levobupivacaine. Black: no siRNA transfection. Black and white: with siRNA transfection, ACE2: angiotensin-converting enzyme 2, BAX: Bcl-2-associated X protein, BCL2: B-cell/CLL lymphoma 2, EGFR: epidermal growth factor receptor, HIF-1α: hypoxia-inducible factor 1α, si: siRNA, WNT1: wingless/Integrated 1.

We performed a molecular biological analysis using RT-PCR to assess the changes caused by levobupivacaine treatment (Figure 3 and Supplementary Table S2). The administration of 0.1 mM levobupivacaine resulted in increased ACE2 expression, whereas the administration of 0.5 mM resulted in equivalent expression to the C group (Figure 3a, relative ratio to C, *n* = 6; C 1.000 ± 0.120 vs. L0.1 1.218 ± 0.041 vs. L0.5 0.703 ± 0.349; C vs. L0.1, *p* = 0.007; C vs. L0.5, *p* = 0.066). Administration of ACE2 siRNA resulted in a 50% decrease in ACE2 expression, confirming that the siRNA design and transfection were appropriate. The administration of levobupivacaine to siRNA-treated groups did not restore ACE2 expression levels comparable to the C group (relative ratio to C, *n* = 6; C 1.000 ± 0.120 vs. si 0.477 ± 0.212 vs. siL0.1 0.702 ± 0.070 vs. siL0.5 0.597 ± 0.068; C vs. si, *p* < 0.001; L0.1 vs. siL0.1, *p* < 0.001; L0.5 vs. siL0.5, *p* < 0.001).

#### 2.2.2. Levobupivacaine Suppressed the Expressions of Cancer Malignancy Markers, but This Suppressive Effect Was Reversed by the Combined Administration of ACE2 siRNA

The levobupivacaine treatment slightly reduced the expression of several markers that are associated with malignancy; however, no significant differences were observed (Supplementary Table S2). In contrast, the combination of ACE2 siRNA with levobupivacaine resulted in an increase in the expression of HIF-1α specifically, negating the changes in expression caused by levobupivacaine (Figure 3b, HIF-1α, relative ratio to C, *n* = 6; C 1.000 ± 0.129 vs. si 0.501 ± 0.072 vs. siL0.1 0.496 ± 0.161 vs. siL0.5 0.464 ± 0.081; C vs. si, *p* = 0.0010; L0.1 vs. siL0.1, *p* < 0.001; L0.5 vs. siL0.5, *p* = 0.1480). As shown in Figure 3c, the administration of levobupivacaine significantly suppressed BCL2 expression at lower concentrations. In contrast, under ACE2 siRNA administration, the BCL2 expression increased remarkably with any concentration of levobupivacaine (Figure 3c, BCL2, relative ratio to C, *n* = 6; C 1.000 ± 0.122 vs. L0.1 0.464 ± 0.099 vs. L0.5 0.992 ± 0.114 vs. si 2.676 ± 0.333 vs. siL0.1 2.129 ± 0.348 vs. siL0.5 2.856 ± 0.412; C vs. L0.1, *p* = 0.0198; C vs. L0.5, *p* > 0.9999; L0.1 vs. L0.5, *p* = 0.0225; C vs. si, *p* < 0.0001; L0.1 vs. siL0.1, *p* < 0.001; L0.5 vs. siL0.5, *p* = 0.1480). The EGFR gene expression showed a similar trend, and it was increased by ACE2 siRNA (Figure 3d, EGFR, relative ratio to C, *n* = 6; C 1.000 ± 0.158 vs. L0.1 0.539 ± 0.182 vs. L0.5 1.113 ± 0.125 vs. si 1.743 ± 0.360 vs. siL0.1 1.726 ± 0.422 vs. siL0.5 1.658 ± 0.454; C vs. si, *p* < 0.001; L0.1 vs. siL0.1, *p* < 0.001; L0.5 vs. siL0.5, *p* = 0.0787). Bax gene expression was slightly reduced at 0.1 mM, and it was increased by the administration of levobupivacaine in combination with ACE2 siRNA (Figure 3e, BAX, relative ratio to C, *n* = 6; C 1.000 ± 0.182 vs. L0.1 0.598 ± 0.121 vs. L0.5 0.784 ± 0.173 vs. si 0.980 ± 0.199 vs. siL0.1 1.224 ± 0.157 vs. siL0.5 1.524 ± 0.284; C vs. L0.1, *p* = 0.0126; C vs. si, *p* > 0.9999; L0.1 vs. siL0.1, *p* < 0.001; L0.5 vs. siL0.5, *p* < 0.0001). WNT1 gene expression was reduced at 0.5 mM levobupivacaine, and it was increased by ACE2 siRNA, which was suppressed when combined with levobupivacaine (Figure 3f, WNT1, relative ratio to C, *n* = 6; C 1.000 ± 0.160 vs. L0.1 1.126 ± 0.214 vs. L0.5 0.318 ± 0.159 vs. si 3.468 ± 0.644 vs. siL0.1 1.673 ± 0.228 vs. siL0.5 0.911 ± 0.292; C vs. L0.1, *p* = 0.0126; C vs. si, *p* > 0.9999; L0.1 vs. siL0.1, *p* < 0.001; L0.5 vs. siL0.5, *p* < 0.0001). The administration of 0.5 mM levobupivacaine significantly reduced β-catenin expression, while siRNA transfection clearly increased it, regardless of levobupivacaine administration (Figure 3g).

### 2.3. Immunofluorescent Analysis

#### 2.3.1. Levobupivacaine Treatment Enhanced the Expression of the Anticancer Marker ACE2

**Figure 4:**
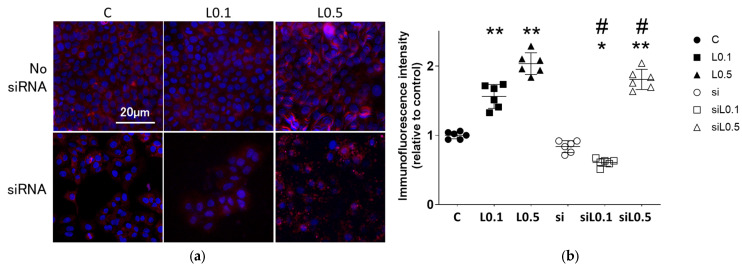
Immunofluorescent images targeting ACE2 (an anticancer factor). (**a**) Representative immunofluorescent images of A549 cells at 24 h after 2 h of levobupivacaine exposure with/without ACE2 siRNA transfection. Blue: DAPI. Red: ACE2. Scale bar: 20 μm, ×20 (**b**) Analysis of the immunofluorescent intensity. Data are mean ± SD. * *p* < 0.05, ** *p* < 0.01 vs. the C group; # *p* < 0.01 vs. the siR-NA-untreated group; *n* = 6, one-way ANOVA followed by a post hoc Tukey test. Dots: no administration of levobupivacaine. Squares: 0.1 mM levobupivacaine. Triangles: 0.5 mM levobupivacaine. Black: no siRNA transfection. Black and white: with siRNA transfection. Scale bar: 20 μm, ×20.

Figure 4a,b provides immunofluorescent images of ACE2 expression changes after 2 h of levobupivacaine exposure with/without ACE2 siRNA transfection. Levobupivacaine increased the expression of ACE2 in a concentration-dependent manner (relative ratio to C, *n* = 6; C vs. L0.1 vs. L0.5, 1.000 ± 0.051 vs. 1.561 ± 0.172 vs. 2.034 ± 0.157, *p* < 0.01). The administration of ACE2 siRNA reduced the expression of ACE2, while the high concentration levobupivacaine enhanced the expression (relative ratio to C, *n* = 6; C vs. si vs. siL0.1 vs. siL0.5, 1.000 ± 0.051 vs. 0.837 ± 0.086 vs. 0.609 ± 0.055 vs. 1.806 ± 0.146, *p* < 0.01). The results of the RT-PCR and immunofluorescence analysis demonstrated that the design and implementation of the ACE2 siRNA were appropriate.

#### 2.3.2. Levobupivacaine Suppressed the Expression of Cancer Malignancy Markers, but the Combined Administration of ACE2 siRNA Reversed This Suppressive Effect

**Figure 5:**
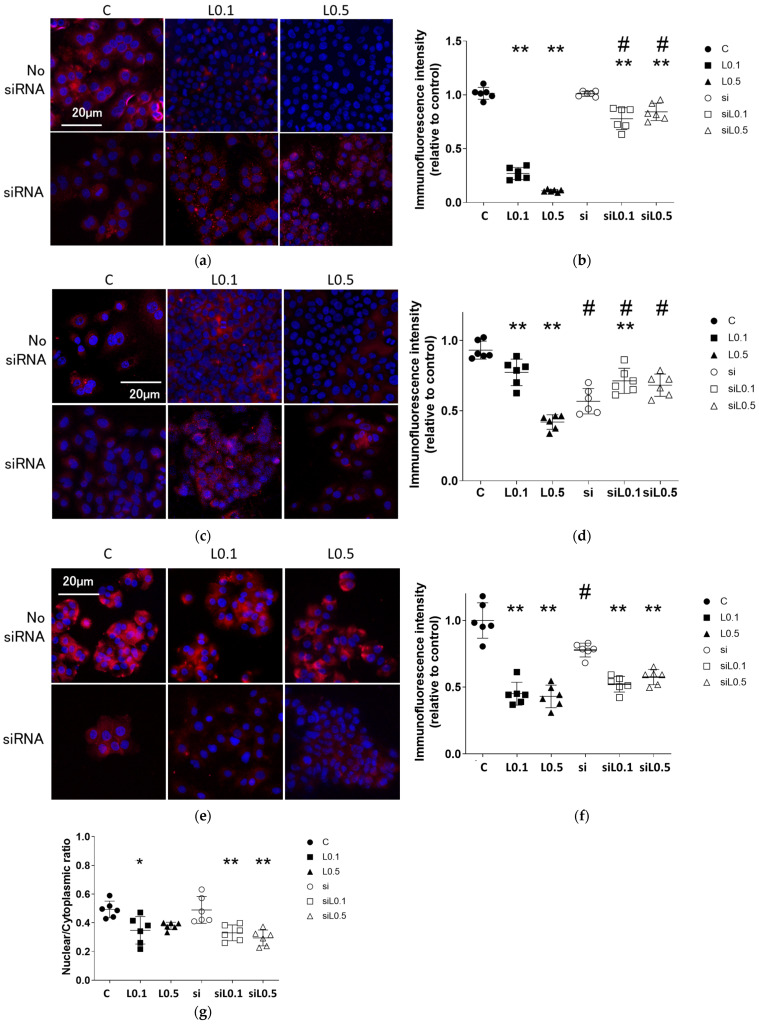
Immunofluorescent images targeting pro-cancer factors. Representative immunofluorescent images of A549 cells at 24 h after 2 h of levobupivacaine exposure with/without ACE2 siRNA transfection. Blue: DAPI. (**a**) HIF-1α (a cancer malignancy marker). (**b**) Analysis of the immunofluorescent intensity of HIF-1α. (**c**) MMP-9 (a metastasis marker). (**d**) The immunofluorescent intensity of MMP-9. (**e**) β-catenin (a cell proliferation promoter). (**f**) The immunofluorescent intensity of β-catenin. (**g**) Analysis of the nuclear/cytoplasmic ratio of β-catenin. Data are mean ± SD. * *p* < 0.05, ** *p* < 0.01 Vs. The C group; # *p* < 0.01 Vs. The siRNA-untreated group (n = 6 samples); one-way ANOVA followed by a Post Hoc Tukey test. Dots: no administration of levobupivacaine. Squares: 0.1 mM levobupivacaine, Triangles: 0.5 mM levobupivacaine. Black: no siRNA transfection. Black and white: with siRNA transfection. C: control (scrRNA-treated), ACE2: angiotensin-converting enzyme 2, HIF-1α: hypoxia-inducible factor 1α, MMP-9: matrix metalloproteinase 9. Scale bar: 20 μm, ×20.

The administration of levobupivacaine slightly reduced the expression of several markers. Figure 5a,b provides immunofluorescent images of the changes in the expression of the cancer malignancy marker HIF-1α after 2 h of levobupivacaine exposure with/without ACE2 siRNA transfection. Levobupivacaine suppressed the expression of HIF-1α in a concentration-dependent manner (relative ratio to C, *n* = 6; C vs. L0.1 vs. L0.5, 1.000 ± 0.055 vs. 0.251 ± 0.054 vs. 0.104 ± 0.027, *p* < 0.01). The administration of ACE2 siRNA enhanced the HIF-1α expression, while high-concentration levobupivacaine slightly decreased it (relative ratio to C, *n* = 6; C vs. si vs. siL0.1 vs. siL0.5, 1.000 ± 0.055 vs. 1.038 ± 0.055 vs. 0.857 ± 0.099 vs. 0.889 ± 0.081, *p* < 0.01). Figure 5c,d shows immunofluorescent images of the changes in the expression of MMP-9 (a metastasis marker) after 2 h of levobupivacaine exposure with/without ACE2 siRNA transfection. Levobupivacaine inhibited the expression of MMP-9 in a concentration-dependent manner, with the following results: C vs. L0.1 vs. L0.5 showed values of 1.000 ± 0.069, 0.838 ± 0.103, and 0.454 ± 0.056, respectively (relative ratio to C, *n* = 6; *p* = 0.019, *p* < 0.0001). The administration of ACE2 siRNA increased the MMP-9 expression. Interestingly, high concentration of levobupivacaine resulted in a slight increase in MMP-9 expression compared to the group that was not treated with siRNA (relative ratio to C, *n* = 6; C vs. si vs. siL0.1 vs. siL0.5, 1.000 ± 0.069 vs. 0.614 ± 0.098 vs. 0.773 ± 0.097 vs. 0.739 ± 0.086, *p* < 0.01). As shown in Figure 5e,f, levobupivacaine inhibited the expression of β-catenin (a cell proliferation promoter) in a concentration-dependent manner, with the following results: C vs. L0.1 vs. L0.5 showed values of 1.000 ± 0.133, 0.451 ± 0.085, and 0.431 ± 0.084, respectively (relative ratio to C, *n* = 6; *p* < 0.0001, *p* < 0.0001). The administration of ACE2 siRNA decreased the β-catenin expression. When ACE2 siRNA was combined with levobupivacaine, β-catenin expression was suppressed, regardless of the concentration administered (relative ratio to C, *n* = 6; C vs. si vs. siL0.1 vs. siL0.5, 1.000 ± 0.133 vs. 0.778 ± 0.052 vs. 0.523 ± 0.059 vs. 0.576 ± 0.058, *p* < 0.01). The immunofluorescent intensity ratio of nuclear and cytoplasmic proteins decreased with levobupivacaine treatment, regardless of whether siRNA transfection was performed with/without siRNA transfection (Figure 5g).

## 3. Discussion

We investigated the effects of levobupivacaine in conjunction with ACE2 knockdown on lung adenocarcinoma A549 cells. Our findings indicate that the administration of levobupivacaine alone did not significantly affect the proliferation of A549 cells, but it significantly reduced the production of ATP, suggesting a greater inhibitory effect on metabolic activity compared to cell proliferation. In contrast, ACE2 knockdown was observed to promote both cell proliferation and ATP metabolism. Importantly, the co-administration of levobupivacaine effectively suppressed these enhancements, suggesting that levobupivacaine may negatively regulate ACE2-dependent proliferation and metabolic activity in lung adenocarcinoma cells. These results contribute to a deeper understanding of the interplay between local anesthetics and metabolic pathways in cancer biology.

Local anesthetics may influence cancer cells’ proliferation and metabolism, but the evidence regarding the role of local anesthetics in ACE2-dependent signaling is limited. One study indicated that treatment with 1 mM levobupivacaine for 5 h promoted the epithelial–mesenchymal transition (EMT) and dissemination in A549 cells [10]. Lidocaine has been reported to inhibit A549 cell proliferation [11] but may also promote migration and resistance to 5-FU [12]. In vivo studies showed that lidocaine inhibits tumor growth in non-small cell lung cancer (NSCLC) models [13]. Ropivacaine has been shown to inhibit proliferation and migration in A549 cells via ACE2-dependent pathways [6] and to reduce cell migration and induce apoptosis in two lung cancer cell types [14]. Our present results demonstrated that exposure to 0.1 mM and 0.5 mM levobupivacaine for 2 h inhibited the metabolism in A549 cells through both ACE2-dependent and independent pathways. The differences between our findings and those of prior investigations may be the result of variations in concentrations and the exposure time. In particular, levobupivacaine did not inhibit cancer cell proliferation, but showed an inhibitory effect on metabolic activity, and the differences from our ropivacaine administration results can be attributed to differences in chemical structure and potency. This highlights the need for further research on the long-term effects of local anesthetics in clinical contexts.

The results of this study demonstrated that levobupivacaine inhibits cellular metabolism, proliferation, and survival signaling pathways (HIF-1α, BCL2, EGFR, and MMP-9) through ACE2-dependent mechanisms, while its effects on the β-catenin pathway are ACE2-independent. ACE2 may act as a tumor suppressor in lung cancer, with higher ACE2 expression linked to improved prognoses in specific cancers, particularly NSCLC [6]. ACE2 expression can vary based on the HIF-1α pathway and treatment environments, influencing the prognosis and drug response [15]. In NSCLC specimens, elevated ACE2 levels correlate with favorable outcomes [16]. In vitro, ACE2 overexpression reduced the survival and migration of lung cancer cells [17] and inhibited angiogenesis [18]. Studies using A549 cells have revealed that ACE2 modulates cell migration, proliferation, and invasion [17]. Integrating our prior research [6] with the present data, the administration of both ropivacaine and levobupivacaine demonstrates that ACE2 functions as an inhibitory factor in A549 cells. However, this study revealed a discrepancy between the genetic changes in ACE2 and the immunofluorescence analysis results. This discrepancy may be due to post-transcriptional modifications by non-coding RNA, or other biomarkers and pathway changes that were not measured in this study. These findings suggest that manipulating ACE2 expression, akin to levobupivacaine’s effects, may offer a promising therapeutic approach for lung cancer.

We examined the roles of HIF-1α, MMP-9, and β-catenin as cancer-promoting factors, and we observed that the levobupivacaine treatment affected the expressions of HIF-1α and MMP-9 in an ACE2-dependent manner, whereas β-catenin was regulated independently. MMP-9 is associated with metastasis and tumor malignancy in various carcinomas [19,20]. HIF-1α enhances mitochondrial metabolism and supports cancer cell survival [21,22], while also promoting MMP-9 expression [23,24,25]. The β-catenin pathway also induces MMP-9 expression [26], with specific interactions between β-catenin and HIF-1α [27,28] linked to glucose metabolism [29,30]. In lung adenocarcinoma, miR-1275 mediates this interaction [31], potentially contributing to metabolic dysfunction and drug resistance [32]. These findings highlight the complex interactions among these factors and their implications for cancer progression and treatment resistance, suggesting the need for further research into their molecular mechanisms. The inhibitory effects of ACE2 on MMP-9 and HIF-1α in lung cancer align with results from several previous studies. It has been observed that ACE2 expression suppresses angiogenesis in A549 cells [18], with MMP-related proteins playing a role downstream [17]. Our earlier research also demonstrated that ACE2 expression negatively regulates both HIF-1α and MMP-9 [6]. The data from our present investigation support these findings.

β-Catenin is a key oncogenic factor in NSCLC, with EGFR mutations known to activate this pathway and promote tumorigenesis [33]. Activation of the Wnt/β-catenin signaling is also implicated in cancer immune evasion and altered metabolic processes [34,35]. Studies using A549 cell lines indicated that ACE2 overexpression affects EMT and may interact with the Wnt pathway [18]. In comparison to ropivacaine administration [6], it was observed that, like ropivacaine, levobupivacaine also suppressed β-catenin expression. Furthermore, ACE2 siRNA administration increased β-catenin expression in both cases, indicating that their effects are quite similar. Our present experiments revealed that levobupivacaine administration significantly reduced total β-catenin expression and decreased the nuclear/cytoplasmic (N/C) ratio. In contrast, siACE2, which reduced 50% of ACE2 expression, did not affect the N/C ratio. These findings suggest that levobupivacaine itself inhibits the Wnt pathway, highlighting the potential of levobupivacaine as a cancer-suppressive agent.

This study has several limitations that need to be addressed. The findings were derived from an in vitro system, and in vivo validation is necessary to confirm the results in a more complex biological context. Further investigations are also needed to assess the concentration dependence of levobupivacaine, its long-term effects of levobupivacaine, and its interactions with other related signaling pathways, such as MAPK and PI3K/Akt. Addressing these factors will provide a more comprehensive understanding of the drug’s pharmacological profile and therapeutic potential. Additionally, the ACE2 overexpression experiments should be completed to confirm the direct relationship between ACE2 and levobupivacaine administration. Despite these limitations, our current findings indicate that perioperative local anesthetics might influence tumor cell metabolism and ACE2-dependent signaling. Understanding ACE2 and its related signaling pathways is crucial for clarifying how anesthetics impact tumor cells. This knowledge may help optimize future treatment strategies and improve perioperative patient management.

## 4. Materials and Methods

### 4.1. Cell Culture

Cells of the human-authenticated lung adenocarcinoma cell line A549 (RIKEN BioResource Research Center, Kyoto, Japan) were cultured in RPMI 1640 medium (Thermo Fisher Scientific [TFS], Tokyo, Japan) containing 10% fetal bovine serum and 1% penicillin/streptomycin (TFS) and maintained in a humidified incubator at 37 °C with a 5% CO_2_ atmosphere.

### 4.2. Anesthetic Administration

Levopubivacaine was obtained from Maruishi Pharmaceutical Co. (Osaka, Japan). The concentrations used for the cell culture treatments were informed by previous research: 0.1 mM and 0.5 mM [10]. A naïve control group was also established, consisting of cells that received no anesthetic treatment. The A549 cells were exposed to each concentration for a duration of 2 h, which approximates the typical length of lung cancer surgical procedures.

### 4.3. siRNA Transfection

siRNA transfection was conducted as described [5]. In brief, cells were transfected with either scrambled siRNA (ScrRNA; referred to as the C group) or an ACE2 siRNA construct at a concentration of 20 nmol/L (see Supplementary Table S1a; Merck Chemicals, Tokyo, Japan; Ajinomoto Bio-Pharma, Osaka, Japan). The transfection procedure used HiPerfect Transfection Reagent (Qiagen, Tokyo, Japan). Six hours later, the transfection solution was replaced with standard culture medium. The siRNA-treated cells were then treated with levobupivacaine for immunostaining and RNA analysis.

### 4.4. Cell Proliferation Test (Cell Counting Kit-8, CCK-8 Assay)

Cells were plated at an approximate density of 5 × 10^3^ cells per well on 96-well plates and allowed to adhere for 24 h. The cells were then exposed to each levobupivacaine concentration or control treatment for 2 h and allowed to sit for another 24 h. The cell counting kit-8 proliferation test was then performed following the manufacturer’s manual (Dojindo Laboratories, Kumamoto, Japan) using a SpectraMax i3x microplate reader (Molecular Devices, Tokyo, Japan).

### 4.5. ATP Analysis

Cells were plated at an approximate density of 5 × 10^3^ cells per well on 96-well plates and allowed to adhere for 24 h. Next, the cells were exposed to each levobupivacaine concentration for 2 h and allowed to sit for another 24 h. An adenosine triphosphate (ATP) analysis was performed using a CellTiter-Glo^®^ 2.0 Cell Viability Assay (Promega, Tokyo, Japan) following the manufacturer’s instructions and the SpectraMax i3x microplate reader.

### 4.6. RNA Extraction

Total RNA was isolated from confluent cells cultured in 60 mm Petri dishes (TFS) following a 2-h exposure to either anesthesia or control conditions, with samples collected 6 h post-treatment. The extraction process used an RNeasy Mini Kit^®^ along with a QIAshredder column (both from Qiagen) according to the manufacturer’s protocols. The quantity and quality of the extracted RNA were evaluated using a NanoDrop microvolume spectrophotometer (TFS). Samples exhibiting an A260/A280 ratio > 1.8 were deemed adequate for subsequent analyses. For further experimentation, 1 mg of each total RNA sample was converted into cDNA with the use of a high-capacity cDNA reverse transcription kit (TFS).

### 4.7. qRT-PCR

We conducted a quantitative reverse transcription polymerase chain reaction (qRT-PCR) analysis on several representative genes: ACE2, HIF-1α, BCL2, EGFR, BAX, WNT1, and β-catenin (Supplementary Table S2). The reactions used TaqMan primers and TaqMan Fast Advanced Mastermix (TFS), and were analyzed using QuantStudio^®^ 5 software (TFS). We selected glyceraldehyde-3-phosphate dehydrogenase (GAPDH) mRNA as the endogenous control to normalize the expression data. The Taqman primers are listed in Supplementary Table S1b. The relative expression ratios were calculated using the comparative 2^−ΔΔCT^ method. The data analyses were performed with Expression Suite software ver. 1.3 (TFS), ensuring an accurate quantification of the gene expression levels.

### 4.8. Immunofluorescence Study

At 24 h after seeding 3 × 10^5^ cells onto 13 mm coverglasses in each well of a 24-well plate, the cells were treated with levobupivacaine at the above-specified concentrations for 2 h, then allowed to rest for an additional 24 h. The cells were fixed using 4% paraformaldehyde and blocked with 10% normal donkey serum (Merck Chemicals, Amsterdam, The Netherlands). Overnight incubation at 4 °C was performed with the following primary antibodies: rabbit anti-ACE2 (1:200; Abcam, Tokyo, Japan), rabbit anti-HIF-1α (1:200; Novus Biologicals, Centennial, CO, USA), rabbit anti-MMP-9 (1:200; Cell Signaling Technology, Tokyo, Japan), and rabbit anti-β-catenin (1:200; Cell Signaling Technology). The samples were then treated with a conjugated secondary antibody (Alexa Fluor^®^ 568; TFS) and co-stained using Vectashield mounting medium containing DAPI (TFS). For imaging, six randomized areas from each slide were selected and examined under a microscope (BX53; Olympus, Tokyo, Japan) using 40× magnification (DP74; Olympus). The image analysis was conducted using the ImageJ 1.54i software.

### 4.9. Statistical Analysis

All numerical data are presented as the mean ± standard deviation (SD) and visualized by the creation of scatter plots. To ensure sufficient statistical power, a sample size of *n* = 6 was calculated as necessary to detect a 30% change with 80% power at a 5% significance level. We performed a one-way analysis of variance (ANOVA) followed by Post Hoc Tukey–Kramer testing with Prism 9.0 software (GraphPad Software, San Diego, CA, USA). In all experiments, *p*-values < 0.05 were deemed significant.

## 5. Conclusions

The administration of levobupivacaine to A549 cells did not significantly affect cell proliferation but led to a reduction in ATP production, indicating a decrease in metabolic activity. In contrast, knocking down ACE2 enhanced both cell proliferation and ATP metabolism. However, when levobupivacaine was co-administered, it suppressed these effects, suggesting that it may negatively regulate ACE2-dependent processes in lung adenocarcinoma cells. These findings provide insight into the interaction between local anesthetics and cancer metabolism.

## Data Availability

The original contributions presented in this study are included in the article/Supplementary Materials. Further inquiries can be directed to the corresponding author.

## Supplementary Materials

The following supporting information can be downloaded at: https://www.mdpi.com/article/10.3390/ijms262210833/s1.
